# Identification of predictive variables for the recurrence of oral mucocele

**DOI:** 10.4317/medoral.22690

**Published:** 2019-03

**Authors:** Yun-Jeong Choi, Jin-Seok Byun, Jae-Kap Choi, Jae-Kwang Jung

**Affiliations:** 1 Department of Oral Medicine, School of Dentistry; 2 IHBR, Kyungpook National University, Daegu, Korea

## Abstract

**Background:**

Oral mucocele is the most common minor salivary gland lesion with good prognosis after surgical removal. However, its recurrence is not rare, sometimes bothersome. This study aimed to identify the possible predictive variables affecting the recurrence rate of oral mucocele.

**Material and Methods:**

The histoclinical data of 164 patients diagnosed with oral mucocele were retrospectively obtained by reviewing dental records. The predictive variables for its recurrence were identified by analyzing its recurrence rate according to clinical variables.

**Results:**

The recurrence rate showed the significant differences according to location and age. Oral mucocele recurred with significantly higher frequency on the ventral mucosa of tongue (50.0%) than on the labial/buccal mucosa (8.8%). Its recurrence was significantly more common in the younger patients (aged < 30 years, 16.0%) than in the older patients (aged > 30 years, 4.4%). However, there was no significant difference in recurrence rates between surgical procedures using scalpels and those using lasers.

**Conclusions:**

Patients with oral mucocele should be more carefully informed of its possible recurrence, especially when it is found on the ventral surface of the tongue or in a younger population.

** Key words:**Age, laser, oral mucocele, recurrence, tongue.

## Introduction

Oral mucocele (OM) is a common exophytic lesion caused by salivary accumulation resulting from pathological changes in oral minor salivary glands (MSGs) (1). It clinically manifests as single or multiple, soft, smooth, spherical, painless nodules, ranging in color from translucent blue to pink (1).

Histologically, OM can be divided into two types, the more frequent extravasation type and the retention type (2). The extravasation type is caused by salivary mucus accumulation in the tissues without epithelial lining. Conversely, the retention type is occasionally found as a cyst lined by epithelium (2). While its etiology remains to be clearly determined, OM has been considered secondary to mechanical trauma and plug formation within the salivary ducts (1,3).

Treatment modalities for OM include surgical excision, marsupialization, cryosurgery, and steroid injection (1). Even though complete surgical excision using conventional scalpels or lasers remains the best treatment approach, the recurrence of OM is not rare. However, few reports have tried to determine the predictive variables for the recurrence of OM (4,5).

Therefore, this study aimed to analyze the clinical characteristics and recurrence rate of OM. Further analysis was finally undertaken to identify the possible predictive variables affecting its recurrence rate.  

## Material and Methods

This study was based on the data obtained from 164 patients (88 men and 76 women; mean age, 24.5 ± 14.3 years) who were diagnosed with OM after visiting Kyungpook National University Dental Hospital from January 2011 to December 2017. The diagnosis for each patient was determined by both clinical and histological examinations through excisional biopsy using a conventional scalpel or laser. Cases with mucoceles on the floor of the mouth were excluded to rule out the possibility of a ranula, which is often regarded as a distinctive disease entity. Surgical procedures were performed as follows: the entire lesion was resected together with the adjacent MSGs using a scalpel (mainly, a No. 15 blade) or CO2 laser (mainly, 3W, in continuous mode) under local anesthesia induced using 2% xylocaine. The wound was sutured to secure hemostasis after conventional surgery. Patients were instructed to return for further examination if the healing was incomplete or if there was any recurrence. They were usually reviewed after several days, a couple of weeks, and 1 month postoperatively.

Clinicopathologic parameters were retrospectively obtained from the clinical database under the approval of the institutional review board (KNUDH-2018-03-003). The following variables were analyzed to determine the clinical features of OM ( Table 1, Table 2): gender, age, location, size, color, surface, the presence of hyperkeratotic change, duration, recurrence, surgical procedure, histological subtype, and clinical department. The recurrence rate was thereafter analyzed to determine its differences depending on different clinical profiles and surgical procedures. Several clinical profiles were re-categorized for statistical analysis; the tongue ventral side and the labial/buccal mucosa was considered as the locations, the pink and white/blue was considered as the colors, age distribution included: <30 and ≥ 30 years, and size included: <10 mm, ≥ 10 mm, duration distribution included: <6 months, ≥ 6 months.

**Table 1:**
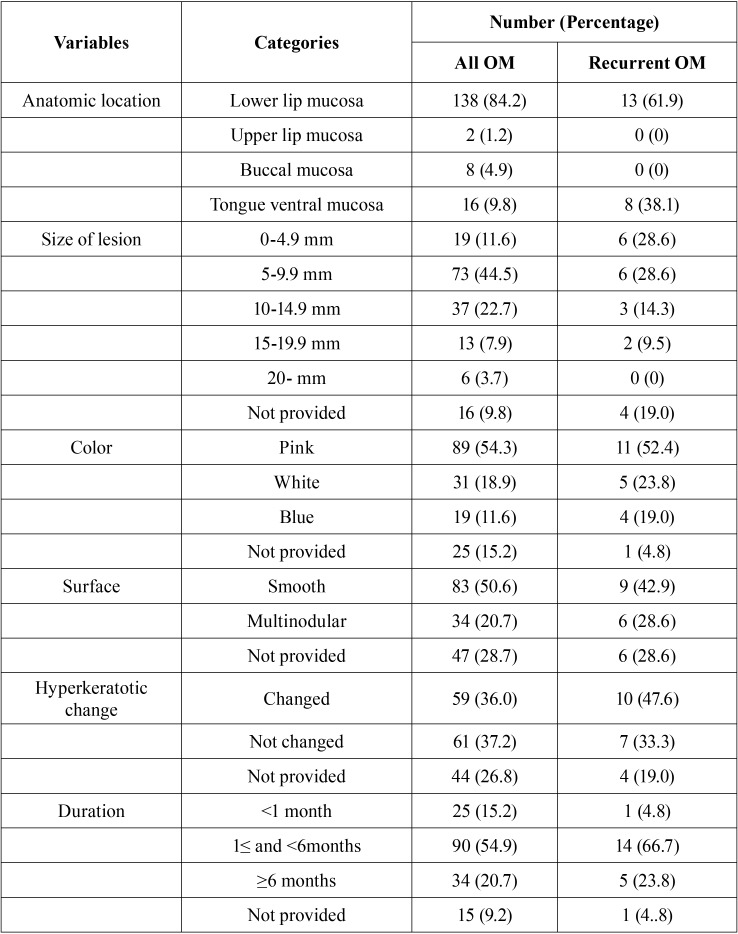
Clinical characteristics of oral mucoceles (OMs).

**Table 2:**
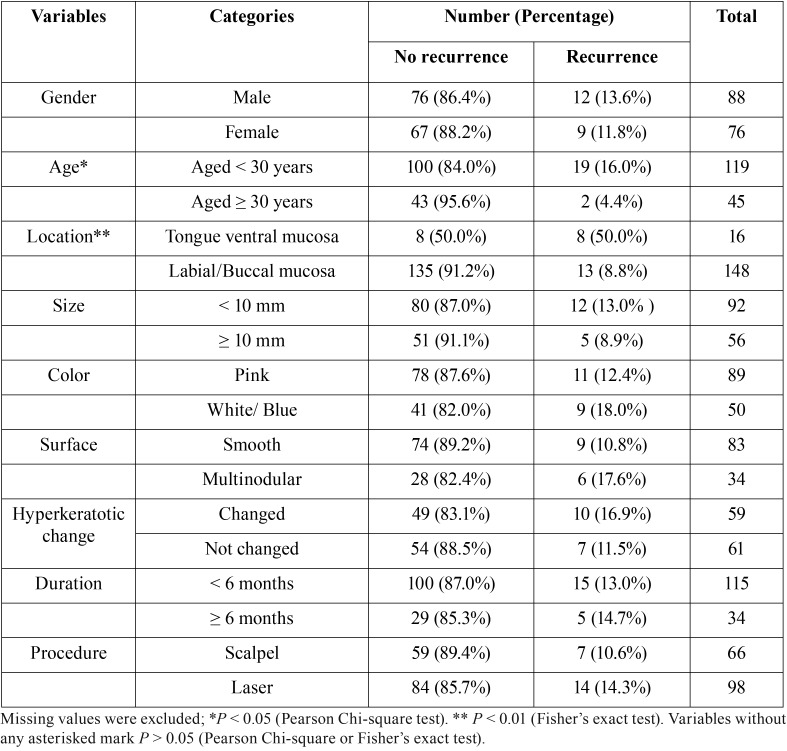
The comparison of recurrence rate according to clinical variances.

Statistical analysis of quantitative variables was performed using descriptive measures, including mean ± standard deviation. Qualitative variables were analyzed by calculating the absolute and relative frequencies (percentages). Descriptive and comparative statistical analyses were performed using the χ2 or Fisher’s exact test for comparing the differences in recurrence rate depending on clinical profiles and surgical procedures. A two-tailed *P*-value < 0.05 was considered statistically significant. All statistical analyses were performed using SPSS 12.0 software for Windows (SPSS Inc, Chicago, IL).

## Results

This study population comprised 164 patients diagnosed with OM; their demographic characteristics are outlined in  Table 1. Most lesions were presented in the first four decades of life (145 cases, 88.4%), with a peak age of 20–29 years old (56 cases, 34.2%), followed by 10–19 years (38 cases, 23.2%). Histological examination revealed that almost all the cases of mucocele were of the extravasation type and only one case was classified as the retention type. Furthermore, most OMs (138 cases, 84. 2%) were located in the lower lip mucosa, followed by the ventral mucosa of the tongue (16 cases, 9.8%), buccal mucosa (8 cases, 4.9%), and upper lip mucosa (2 cases, 1.2%). In most cases (73 cases, 44.5%), the sizes ranged from 5 to 9 mm for the long axis, with the largest being approximately 40 mm. In almost half of the cases (89 cases, 54.3%), the lesion was pink, while the others were pale white (18.9%) and blue (11.6%). In half of the cases (83 cases, 50.6%), the surface was smooth, while multinodular surface was found in 34 cases (20.7%). Duration of the lesion varied, as our results showed that most patients visited within one to six months after identifying the lesion ( Table 1).

Regarding treatment and recurrence, all cases underwent surgical procedures using conventional scalpel (66 cases, 40.2%) or laser (98 cases, 59.8%). Treatment was commonly performed in the departments of oral medicine (99 cases, 60.4%), oral and maxillofacial surgery (52 cases, 31.7%), and pediatric dentistry (13 cases, 7.9%). Recurrence was found in 21 cases (12.8%), being most common in the first month following surgical removal (12 cases, 57.1%), while the most delayed recurrence occurred after 41 months.

Analysis of recurrence rate according to clinical profiles revealed that there were significant differences only depending on the anatomic location and age distribution: ventral mucosa of the tongue (50%, 8 cases of 16 cases) versus labial/buccal mucosa (8.8%, 13 cases of 148 cases); aged < 30 years (16.0% 19 cases of 119 cases) versus ≥ 30 years (4.4% 2 cases of 45 cases) ( Table 2). Recurrence rate according to surgical procedure was slightly higher, but not significantly so, in cases with laser surgery (14.3%, 14 cases of 98 cases) than those with conventional surgery (10.6%, 7 cases of 66 cases). 

## Discussion

Mucoceles are the most common benign minor salivary gland disorders and the second-most common benign soft tissue masses in the oral cavity, after focal fibrous hyperplasia (6,7). Our study showed that most OMs manifested as smooth, pink swellings, a finding consistent with previous ones (8). The analysis of age distribution revealed that OMs were most prevalent among young adults aged 20-29 years (34.2%), followed by the adolescent age group of 10-19 years (23.2%). Similarly, some studies have reported that the teenaged population comprised the most prevalent age group for OMs, while other studies have reported young adulthood as the most prevalent period (3,9,10). These age distributions might be related to the higher prevalence of para-functional oral habits during adolescence and young adulthood. A previous study reported that cheek- and lip-biting are the most prevalent causes of lesions in children and young adults in the United States (11). Furthermore, it also revealed that the lip was the most common site for lesions (11).

 Our study also showed that the most frequent site of OM was the lower lip mucosa, which was similar to the findings described in previous studies (2,4). This can be mainly explained by the fact that the lower lip is one of the oral sites most vulnerable to trauma during parafunctional or functional activities, considering that the lower lip moves dynamically during mastication and speech (12). Another explanation is the varied distribution of MSGs according to anatomic locations (13). Previous studies revealed that the density of glandular areas was significantly greater in the lower lip than in the upper lip (13,14). The second-most common site of OMs was found to be the ventral mucosa of the tongue. Previous studies have reported varying degree of involvement of the tongue in OMs (2,4,9,10). A previous study reported that the tongue area accounted for only nine (2.25%) out of a total of 400 mucocele cases, while another study reported a more frequent involvement of the tongue (17%), which is similar to our finding (2,10). It has been established that the tongue has distinctive types of MSGs, unlike other locations of the oral cavity, possibly leading to different clinical profiles and recurrence patterns of OMs (15).

Our study found that the overall recurrence rate of OMs was slightly higher (12.8%) than those reported in other studies. Previous studies have reported varying recurrence rates, ranging from 2.8% to 18% (2,4,5,9). Interestingly, a previous study with a higher percentage of tongue mucoceles (17%) also showed the markedly increased recurrence rate of 18% (2). Our analysis revealed that the recurrence rate was significantly higher in the ventral mucosa of the tongue (50%) than in the labial/buccal mucosa (8.8%). We thought that this might be mainly because of the probable involvement of the Blandin-Nuhn glands in the occurrence of mucoceles on the ventral mucosa of the tongue. In human tongues, there are three types of MSGs: Weber, von Ebner, and Blandin-Nuhn glands (15). The Weber glands are located along the border of the lateral tongue, while von Ebner glands are found in the trough surrounding the circumvallate papillae on the posterodorsal mucosa of the tongue. Lastly, Blandin-Nuhn glands are mainly distributed on the anteroventral mucosa of the tongue, at a depth of approximately 12-25 mm (15,16). Their deep position might make the complete excision of the causal glands more difficult possibly due to poor view of the operative field. It was also reported that mucoceles on the anteroventral surface of the tongue were usually lined by thinner walls, indicating the possibility of easier rupture during removal (16). Sudden rupture would also cause loss of anatomical references and subsequent ambiguity of the boundaries, thereby making it difficult to ascertain the complete removal of the mucocele (4).

In addition, our study showed that OMs recurred more frequently, with statistically significant difference, in the younger patients (aged < 30 years, 16.0%) than in the older patients (aged ≥ 30 years, 4.4%). Numerous reports stated the considerable prevalence of various oral habits, including lip/cheek biting and tongue thrusting, especially in childhood and young adolescent period (17-19). However, we found no significant difference in the recurrence rate between surgical procedures using lasers and those using scalpels, while a previous study showed that conventional excision using scalpels resulted in a higher recurrence rate than laser excision (4). However, another study reported that the recurrence rate was not significantly different between these surgical procedures (20). Therefore, despite the growing popularity of laser, the advantage of lasers in preventing the recurrence of OM still remains to be carefully determined.

However, there are several inherent limitations to the present study, mainly due to its retrospective design. Firstly, a considerable portion of the information was missing in some cases, and the recurrence rates were not determined based on regular follow-up, while the patients were sufficiently instructed on the possibility of recurrence and the requirement of a re-visit if OM-like lesions recur.

In summary, OM is a common exophytic lesion originating from obstructed MSGs, mainly found on the lower labial mucosa of younger people. The recurrence rate of OM found on the ventral surface of the tongue was much higher than those in other areas. Its recurrence was also more common in the younger patients, including children and teenagers. Therefore, it is recommended that patients with mucoceles, especially younger patients with OMs on ventral mucosa of the tongue, should be informed of the probability of recurrence and the need for subsequent reoperation.
